# The Crude Skin Secretion of the Pepper Frog *Leptodactylus labyrinthicus* Is Rich in Metallo and Serine Peptidases

**DOI:** 10.1371/journal.pone.0096893

**Published:** 2014-06-06

**Authors:** Michelle da Silva Libério, Izabela M. D. Bastos, Osmindo R. Pires Júnior, Wagner Fontes, Jaime M. Santana, Mariana S. Castro

**Affiliations:** 1 Toxinology Laboratory, Department of Physiological Sciences, Institute of Biology, University of Brasilia, Brasilia – DF, Brazil; 2 Brazilian Center for Protein Research, Department of Cell Biology, Institute of Biology, University of Brasilia, Brasilia – DF, Brazil; 3 Laboratory of Host-Pathogen Interaction, Institute of Biology, University of Brasilia, Brasilia – DF, Brazil; University of Iowa, United States of America

## Abstract

Peptidases are ubiquitous enzymes involved in diverse biological processes. Fragments from bioactive peptides have been found in skin secretions from frogs, and their presence suggests processing by peptidases. Thus, the aim of this work was to characterize the peptidase activity present in the skin secretion of *Leptodactylus labyrinthicus*. Zymography revealed the presence of three bands of gelatinase activity of approximately 60 kDa, 66 kDa, and 80 kDa, which the first two were calcium-dependent. These three bands were inhibited either by ethylenediaminetetraacetic acid (EDTA) and phenathroline; thus, they were characterized as metallopeptidases. Furthermore, the proteolytic enzymes identified were active only at pH 6.0–10.0, and their activity increased in the presence of CHAPS or NaCl. Experiments with fluorogenic substrates incubated with skin secretions identified aminopeptidase activity, with cleavage after leucine, proline, and alanine residues. This activity was directly proportional to the protein concentration, and it was inhibited in the presence of metallo and serine peptidase inhibitors. Besides, the optimal pH for substrate cleavage was determined to be 7.0–8.0. The results of the *in* *gel* activity assay showed that all substrates were hydrolyzed by a 45 kDa peptidase. Gly-Pro-AMC was also cleaved by a peptidase greater than 97 kDa. The data suggest the presence of dipeptidyl peptidases (DPPs) and metallopeptidases; however, further research is necessary. In conclusion, our work will help to elucidate the implication of these enzymatic activities in the processing of the bioactive peptides present in frog venom, expanding the knowledge of amphibian biology.

## Introduction

Anuran skin is source of a large variety of substances with different biological activities, such as biogenic amines, steroids, alkaloids, bufadienolides, peptides, and proteins [1]. Most of these compounds are produced by the granular glands present chiefly in the skin of the dorsal region and are involved in protecting against predators and pathogens [2], [3].

Secretion compositions differ among amphibian groups according to their interactions with the environment. Antimicrobial peptides (AMPs) show an important role in innate immunity, besides being important to angiogenesis, tegument repair, inflammatory processes, and chemotaxis [4]. Many of these peptides present identical or analogous functions to the ones found in extracutaneous tissues, including the central and peripheral nervous systems, and the gastrointestinal tract of all vertebrate classes. Similar peptides are present in both secretions and tissues due to the common embryonic-ectodermal origin of the vertebrates’ skin and brain [5]–[7].

Bioactive peptides are secreted by a holocrine mechanism; while some are constitutively expressed, others are induced by the presence of microorganisms or by endogenous pro-inflammatory cytokines in situations of stress or injury [8]–[11]. The AMPs are derived from proteolytic processing of the precursor and consist of a signal sequence, an acidic pro-peptide domain, and a single copy of the biologically active peptide. The signal portion addresses the precursor to an appropriate location in the gland [12], [13]. When the animal is stimulated, a protease removes the acidic region, liberating the peptide that can undergo post-translational modifications; for example, amidation of the C-terminus or further proteolytic processing can occur [14], [15]. The pre-pro-region is conserved among different species, which reinforces the hypothesis that one encoder exon of a large number of unrelated precursors appeared in the beginning of amphibian evolution [12], [13].

During the last decades, the majority of studies regarding biochemical analysis of anuran skin secretions have focused on the isolation and characterization of bioactive peptides. However, little research has focused on the enzymes responsible for peptide processing. In the late 1980s and early 1990s, several studies described the peptidases found in the cutaneous secretion of *Xenopus laevis* [16]–[19].

Skin secretions from the genus *Leptodactylus* have been a rich source of numerous AMPs discovered in the last few years [20]–[27]. On the other hand, no studies have investigated the peptidases, the enzymes responsible for the peptide processing, in this secretion. Previously, we detected two inactive fragments of the AMP fallaxin in the skin secretion of *Leptodactylus labyrinthicus*, which called our attention to the enzymes involved in these proteolytic cleavages. Other research groups also have identified the presence of AMP fragments in their studies [22], [24], [26]. Therefore, the aim of the present study was to identify and characterize the proteolytic activity present in the crude skin secretion of the pepper frog *L. labyrinthicus*.

## Materials and Methods

### Specimen Collection and Skin Secretion Extraction

Adult specimens of *L. labyrinthicus* were collected in Luziânia, GO, Brazil and were maintained in captivity at the University of Brasilia. The skin secretion was obtained by a mild electrical stimulation method and diluted in Milli-Q water. Part of the collected sample was immediately used; the other part was lyophilized and kept at −20°C for subsequent use. The animals reassumed their normal behavior a few minutes after harvesting the secretion. All procedures were performed under an official licence number 17682-1 from ICMBio (Chico Mendes Institute for Conservation of Biodiversity) and were approved by the Animal Ethics Committee of the University of Brasilia.

Protein content was determined by the Bradford method using bovine serum albumin (BSA; Sigma-Aldrich Company, USA) as the standard protein [28].

### Gelatinase Activity

The gelatinase activity assay was performed following the procedure of Menezes et al. [29] with some modifications. In summary, lyophilized samples (40 µg) of the skin secretion of *L. labyrinthicus* were mixed with semi-native sample buffer (62.5 mM Tris-HCl, pH 6.8, 2% (w/v) sodium dodecyl sulfate (SDS), 15% (v/v) glycerol, and 0.02% (w/v) bromophenol blue). The samples were loaded on a 9% SDS-PAGE gel co-polymerized with 0.1% (w/v) gelatin. To visualize the bands of activity, the gels were submitted to four washes of 15 min each with 2.5% (v/v) Triton X-100 to remove traces of SDS. Next, the gels were washed with deionized water to remove excess Triton X-100, and they were incubated at room temperature for 22 h in different activity buffers as described below. Lastly, the gels were stained with Coomassie Blue R-250 and destained. Hydrolysis bands were visualized as clear bands against a blue background, and were quantified by densitometry using the software ImageJ (U.S. National Institutes of Health - NIH).

To identify the best activity incubation buffer, the following buffers were tested at pH 8.0: 50 mM Tris-HCl; 50 mM Tris-HCl with 1 mM or 10 mM CaCl_2_; 50 mM Tris-HCl, 150 mM NaCl, and 10 mM CaCl_2_; 50 mM Tris-HCl, 150 mM NaCl, 10 mM CaCl_2_, and 0.02% (w/v) 3-[(3-cholamidopropyl)dimethylammonio]-1-propanesulfonate (CHAPS). To study the thermostability of the sample, 40 µg of lyophilized skin secretion was boiled for 5 min before loading onto the gel. Later, it was incubated with the last buffer described.

The pH dependence of the gelatinase activity was studied by incubating the gels in different buffers. The buffers tested were 50 mM sodium acetate, and 10 mM CaCl_2_, pH 4.0–5.0; 50 mM 4-(2-hydroxyethyl)-1-piperazineethanesulfonic acid (HEPES), and 10 mM CaCl_2_, pH 6.0–7.0; and 50 mM Tris-HCl, and 10 mM CaCl_2_, pH 8.0–10.0.

### Effects of Inhibitors in the Gelatinase Activity Assay

Zymograms were performed as described above in the presence of different protease inhibitors. The samples were mixed with sample buffer containing 10 mM ethylenediaminetetraacetic acid (EDTA), 10 mM phenathroline, 100 µM tosyl lysine chloromethyl ketone (TLCK), 10 µM E-64, 1 µM pepstatin A, 1 mM 4-(2-aminoethyl)-benzenesulfonyl fluoride (AEBSF), or 100 µM leupeptin. After washing with 2.5% (v/v) Triton X-100 and deionized water, the gels were incubated at room temperature for 22 h in activity buffer (50 mM Tris-HCl, 150 mM NaCl, and 10 mM CaCl_2_, pH 8.0) containing inhibitors at the same concentrations as described above. The bands were quantified by densitometry using the software ImageJ (U.S. National Institutes of Health - NIH).

### Enzymatic Assays Using Fluorogenic Substrates

The substrate specificity for the pool of peptidases present in the skin secretion of *L. labyrinthicus* was evaluated by measuring the fluorescence of 7-amino-4-methylcoumarin (AMC) released by hydrolysis of fluorogenic substrates as previously described [30]. Briefly, the reactions were carried out by incubation of 10 µL (15.6 µg) of fresh skin secretion, 80 µL of 25 mM HEPES buffer (pH 7.5), and 10 µL of 200 µM N-Succinyl-Gly-Pro-Leu-Gly-Pro-AMC, Gly-Pro-AMC, N-Succinyl-Ile-Ala-AMC, l-Pro-AMC, N-Succinyl-Leu-Leu-Val-Tyr-AMC, l-Leu-AMC, N-Succinyl-Leu-Tyr-AMC, Nα-CBZ-Gly-Gly-Arg-AMC, N-CBZ-Gly-Gly-Arg-AMC, N-CBZ-Phe-Arg-AMC, l-Alanyl-l-Alanyl-l-Phe-Ala-AMC, or Nα-CBZ-l-Arg-AMC. As control, buffer was used instead of the fresh skin secretion sample. The fluorescence of the AMC released was recorded up to 30 min at 460 nm upon excitation at 355 nm using a microplate reader (SpectraMax M2, Molecular Devices, USA). The rate of each reaction was calculated by Soft Max Pro software (Molecular Devices, USA) in milli units of fluorescence liberated per minute (mUF/min). The assay was performed in triplicate.

### Influence of Protein Concentration on Proteolytic Activity

A fluorogenic enzymatic assay was carried out as described above using different concentrations of the sample: 0, 2, 4, 8, 16, 32, and 64 µL (1.25 µg/µL) with 20 µM substrate (Gly-Pro-AMC, l-Leu-AMC, or l-Alanyl-l-Alanyl-l-Phe-Ala-AMC).

### Determination of pH Dependence

The assay was performed by incubating 32 µL (1.25 µg/µL) of the fresh sample with 58 µL of AMT buffer (100 mM acetic acid, 100 mM MES, and 100 mM Tris-base) adjusted to pH 5.0 to 10.0 at 25°C for 15 min. After this time, 10 µL of 200 µM Gly-Pro-AMC, l-Leu-AMC, or l-Alanyl-l-Alanyl-l-Phe-Ala-AMC was added to the reaction; and AMC release was recorded as above.

### Effects of Inhibitors in the Fluorogenic Assay

Aliquots of 32 µL (1.25 µg/µL) of fresh skin secretion were incubated with 10 µL of different inhibitors for 45 min at 25°C before addition of 20 µM substrate (Gly-Pro-AMC, l-Leu-AMC, or l-Alanyl-l-Alanyl-l-Phe-Ala-AMC). Each substrate was tested with each inhibitor at the following final concentrations: 100 µM tosylamide-2-phenylethylchloromethyl ketone (TPCK), 1 mM phenylmethanesulphonyl fluoride (PMSF), 100 µM TLCK, 1 µM pepstatin A, 100 µM leupeptin, 10 mM EDTA, 10 µM E-64, 10 µM bestatin, and 10 mM phenanthroline. The inhibitors were prepared according to the manufacturer’s recommendations. Control reactions were performed in the presence of the solvent used to dilute the inhibitors, under the same conditions. Inhibition rate was expressed as relative activity compared to the control.

### In-gel Activity

Proteolytic activity in SDS-PAGE was performed as described [31]. Briefly, six aliquots of 40 µg of the crude skin secretion of *L. labyrinthicus* were mixed with semi-native sample buffer (three of them were boiled for 5 min) and resolved on 9% SDS-PAGE at 4°C. After electrophoresis, the gels were washed four times for 15 min each with 2.5% (v/v) Triton X-100 and with 25 mM HEPES, pH 7.5. Finally, the gels were incubated at 25°C for 15 min with 20 µM fluorogenic substrate (Gly-Pro-AMC, l-Leu-AMC, or l-Alanyl-l-Alanyl-l-Phe-Ala-AMC), and the activity bands were documented under ultra-violet exposure. One of the gels was later stained with silver nitrate [32]. The activity bands were quantified by densitometry using the software ImageJ (U.S. National Institutes of Health - NIH).

## Results and Discussion

The identification of two fragments of the peptide fallaxin in the skin secretion of *L. labyrinthicus* instigated our group to study the secreted proteolytic enzymes responsible for peptide processing in the skin of this anuran. Truncated peptides also have been found in the skin secretion of other *Leptodactylus* species. Leite et al. [26] identified five truncated forms of ocellatins from *L. ocellatus*. Dourado et al. [24] found six fragments of the peptide syphaxin from *L. syphax*. In addition, a 22-residue fallaxin fragment identified by our group was isolated by Rollins-Smith et al. [22] from the skin secretion of *L. fallax* as well. According to Giovannini et al. [16], lysis of secretory vesicles could expose mature peptides to extravesicular proteolytic enzymes capable of cleaving these peptides into fragments.

### Gelatinase Activity

**Figure 1:**
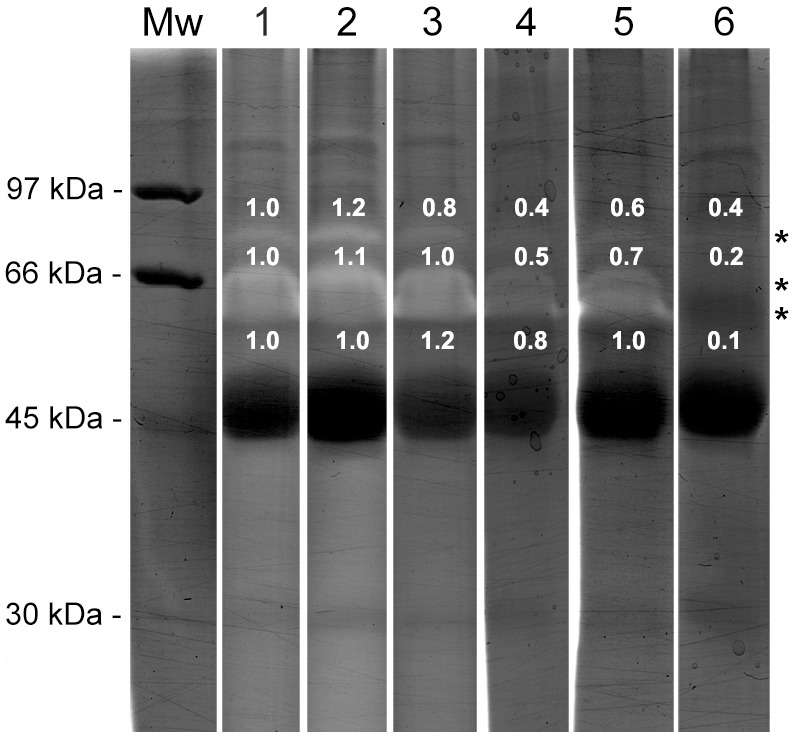
Gelatinase activity of the crude skin secretions of *L. labyrinthicus*. Sample (40 µg) was separated by 9% SDS-PAGE co-polymerized with 0.1% gelatin. The gels were incubated for 22 h with the following buffers at pH 8.0: (lanes 1 and 2) 0.02% (w/v) CHAPS, 50 mM Tris-HCl, 150 mM NaCl, and 10 mM CaCl_2_; (lane 3) 50 mM Tris-HCl, 150 mM NaCl, and 10 mM CaCl_2_; (lane 4) 50 mM Tris-HCl and 1 mM CaCl_2_; (lane 5) 50 mM Tris-HCl and 10 mM CaCl_2_; (lane 6) 50 mM Tris-HCl. Lane 1 was loaded with native crude skin secretions. The same sample was previously boiled for 5 min before loading in lane 2. The bands were quantified by densitometry and the values were normalized in relation to lane 1. The values are shown close to their respective bands. Asterisks indicate bands with molecular weights of approximately 60, 66, and 80 kDa. Mw: molecular weight markers.

In our search for peptidase activity in the crude skin secretion of *L. labyrinthicus*, we identified proteolytic activity on gelatin gels. The gelatinase activity was visualized by zymography after loading 40 µg of lyophilized crude secretion onto a 9% SDS-PAGE gel co-polymerized with 0.1% (w/v) gelatin. A fresh sample containing the same protein concentration was also analyzed; however, the proteolytic activity observed did not differ from the lyophilized one (data not shown). The zymogram revealed the presence of three gelatinolytic bands of approximately 60, 66, and 80 kDa (Fig. 1). More accurate molecular weights of the enzymes could not be determined because the samples were not denatured by heat treatment or reduced to prevent protein-protein aggregation; moreover, the presence of gelatin can affect protein mobility [33].

This gelatinolytic assay is highly sensitive, detecting picomolar levels of enzymes. Furthermore, it also separates proteolytic enzymes from activity-masking agents, such as inhibitors and substrates that could be present in the skin secretion [34]. The gelatinase activity may play a role in wound healing in amphibians, as suggested to some AMPs. Fibroblast activation protein (FAP), a 170 kDa protein, is an example of an enzyme that cleaves gelatin and is also capable of modifying bioactive peptides; in addition, it is involved in wound healing in humans [35].

Therefore, the gelatinase activity of the lyophilized sample was analyzed using different activity buffers to determine the best conditions. The 60 and 66 kDa bands were only detected on the gel incubated with buffer containing CaCl_2_, demonstrating that the proteolytic activity of these bands is calcium dependent. It was also noticed that the increase of CaCl_2_ concentration from 1 mM to 10 mM increased the proteolysis. The presence of CHAPS or NaCl improved the activities of the 60, 66, and 80 kDa bands. CHAPS was considerably important to improve the activity of the 80 kDa band. Nevertheless, its absence contributed to the gelatinase activity of the 60 kDa band and did not interfere in the activity of the 66 kDa band. Some detergents can interfere with peptidase activity. Sah and Kim [36] suggested that CHAPS can modulate the activity and stability of some proteins. Our results suggest that the highest activity observed in the presence of NaCl or CHAPS was achieved due to the increase of the ionic strength of the incubation buffer.

The thermostability of the peptidases present in the skin secretion of the pepper fog is noteworthy. Even after being boiled for 5 min, the sample preserved its gelatinase activity (Fig. 1, lane 2). The presence of an active enzyme at elevated temperatures has already been reported in ectothermic vertebrates. Merchant and collaborators [37] identified dipeptidyl peptidase 4 (DPP4) activity in the serum of the American crocodile with increased activity at higher temperatures. A peptidase A isolated from the venom of *Bothrops jararaca* also showed to be stable at high temperatures and at pHs between 3.0 and 9.0 [38]. This serine peptidase that hydrolyses gelatin is highly glycosylated. There are evidences that glycosylation increases the stability of proteases [39]. In the case of the *Bothrops* peptidase A its sugar moiety also probably avoided the interaction of many inhibitors with the polypeptide core of the enzyme [40]. Thus it is possible that the termostability of the peptidases found in the skin secretions of *L. labyrinthicus* is due to the presence of carbohydrate moieties in their structure. Since the physiological processes of ectothermic vertebrates are dependent on environmental temperatures, it is expected that some of their enzymatic activities are temperature dependent.

**Figure 2:**
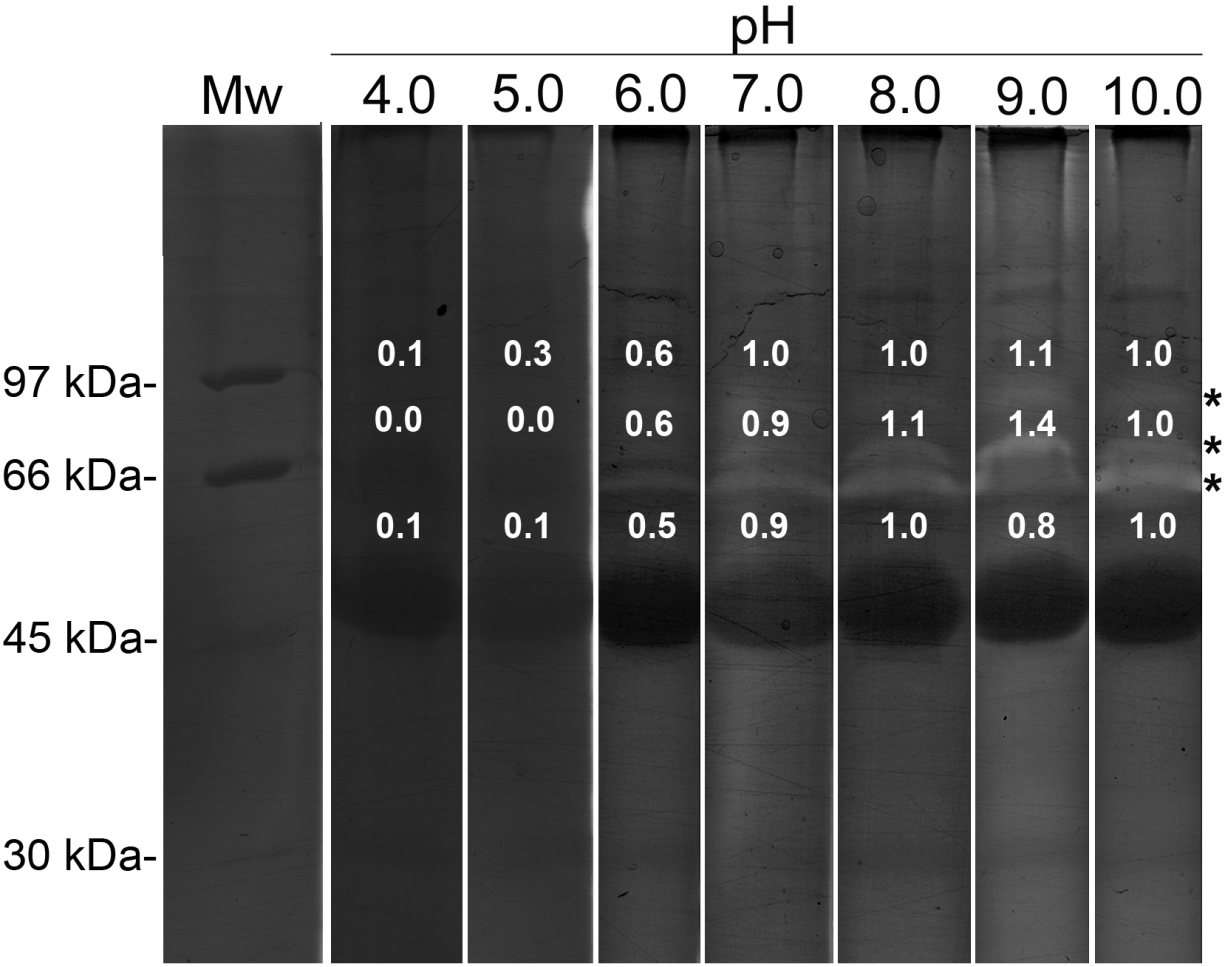
Analysis of the pH dependence for the gelatinase activity present in the crude skin secretions of *L. labyrinthicus*. The gels (9% SDS-PAGE/gelatin) were incubated for 22 h under the following conditions: 10 mM CaCl_2_, 50 mM sodium acetate, pH 4.0–5.0; 10 mM CaCl_2_, 50 mM HEPES, pH 6.0–7.0; and 10 mM CaCl_2_, 50 mM Tris-HCl, pH 8.0–10.0. The bands were quantified by densitometry and the values were normalized in relation to pH 10.0 gel. The values are shown close to their respective bands. Mw: molecular weight markers.

In an attempt to determine the pH dependence, it was shown that the 60, 66, and 80 kDa bands had gelatinase activity in a broad pH range from 6 to 10 and that these bands were practically inactive below pH 6. The 60 kDa band was more active at pH 9.0 (Fig. 2).

**Figure 3:**
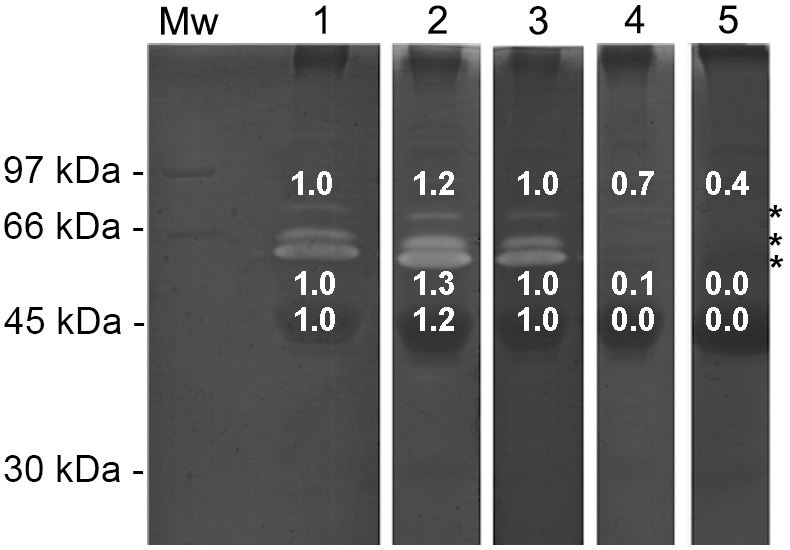
Effects of different inhibitors on the gelatinase activity of the crude skin secretions of *L. labyrinthicus*. The samples (40 µg) were mixed with the following inhibitors in sample buffer (50 mM Tris-HCl, 150 mM NaCl, and 10 mM CaCl_2_, pH 8.0) before loading onto the 9% SDS-PAGE/gelatin gel. Lane 1: no inhibitor; lane 2: 0.1 mM TLCK; lane 3: 0.01 mM E-64; lane 4: 10 mM phenathroline; lane 5: 10 mM EDTA. The bands were quantified by densitometry and the values were normalized in relation to the sample control which has no inhibitor treatment (lane 1). The values are shown close to their respective non-stained bands. Mw: molecular weight markers.

In order to define the classes of the peptidases identified in the zymogram, different protease inhibitors were added to the sample buffer and to the incubation buffer, and their effects were examined. In the presence of EDTA or phenanthroline (Fig. 3) complete inhibition of the 60, and 66 kDa gelatinolytic bands was observed. These data, together with the calcium-dependent activity and the pH profile, indicate the presence of metallopeptidases in these gelatinolytic bands [41]. The 80 kDa band was also inhibited by EDTA and slightly by penanthroline (Fig. 3), however this band was not inhibited by inhibitors of serine, cysteine or aspartic (data not shown), what reinforces that it is a metallopeptidase. The peptidase activity of the 60 kDa band was also slightly reduced by pepstatin, AEBSF and leupeptin, inhibitors of aspartic, serine, and cysteine/serine proteases with trypsin-like activity, respectively (data not shown). Despite of the weak inhibition by pepstatin the data do not suggest the presence of aspartic peptidases since no cleavage of gelatin was observed at the optimum pH for these enzymes (acidic).

### Specificity of the Peptidases

Aiming to identify the specificity of the peptidases present in the sample, 15.6 µg of fresh skin secretion was incubated with twelve different fluorogenic peptides. Only three peptides were hydrolyzed by the sample: Gly-Pro-AMC, l-Leu-AMC, and l-Alanyl-l-Alanyl-l-Phe-Ala-AMC. All the hydrolysis occurred after an apolar amino acid residue. Similar results were found in a study of the metallopeptidase dactylysin purified from a *Xenopus laevis* skin secretion, which cleaves between doublets of hydrophobic amino acids [19].

The highest proteolytic activity was against Gly-Pro-AMC, and the lowest activity was observed with l-Alanyl-l-Alanyl-l-Phe-Ala-AMC (Table 1). Cleavage after proline residues is a characteristic of prolyl peptidases (serine peptidases of family S9), which are enzymes that play a major role in the regulation of many biological functions through the processing of peptide hormones, as proline is not cleavable by many cellular proteases *in vivo* [30]. Moreover, only the peptides with unsubstituted amino termini were cleaved by the peptidases found in the studied venom, which indicates the presence of aminopeptidases; for instance, the peptide N-Succinyl-Gly-Pro-Leu-Gly-Pro-AMC was not hydrolyzed. Several DPPs are prolyl peptidases that are able to hydrolyze different dipeptide sequences from the amino termini of peptides. This subfamily (S9B) includes DPP4, FAP, DPP8, and DPP9 [42]. The hypothesis of hydrolysis by dipeptidases is supported by the observation that the substrate L-Pro-AMC was not cleaved when incubated with the crude secretion.

**Table 1:** Specificity of the peptidases present in the crude skin secretions of *L. labyrinthicus* on various fluorescent synthetic substrates.

Substrate	Activity (mUF/min)	Relative activity^a^ (%)
Gly-Pro-AMC	23.17	100.0
l-Leu-AMC	21.55	93.0
l-Alanyl-l-Alanyl-l-Phe-Ala-AMC	8.34	36.0
Nα-CBZ-l-Arg-AMC	0.99	4.3
N-Succinyl-Ile-Ala-AMC	0.44	1.9
l-Pro-AMC	0.41	1.8
N-Succinyl-Leu-Leu-Val-Tyr-AMC	0.10	0.4
N-Succinyl-Leu-Tyr-AMC	0.05	0.2
N-Succinyl-Gly-Pro-Leu-Gly-Pro-AMC	0.00	0.0
Nα-CBZ-Gly-Gly-Arg-AMC	0.00	0.0
N-CBZ-Gly-Gly-Arg-AMC	0.00	0.0
N-CBZ-Phe-Arg-AMC	0.00	0.0

a Expressed as a percentage of Gly-Pro-AMC hydrolyzing activity, which was taken as 100%.

Our search in the online peptidase database *MEROPS* (merops.sanger.ac.uk) found 16 hits for peptidases able to cleave l-Leu-AMC and did not return any hits with l-Alanyl-l-Alanyl-l-Phe-Ala-AMC [43]. The presence of aminopeptidases that cleave after alanine and leucine in the skin secretion of an anuran was also identified by Giovannini et al. [16]. In their work, cleavage sites at the N-terminal side of Xaa-Lys residues, where Xaa is Leu, Gly, Ala, or Lys, were observed. Metallopeptidases and cysteine peptidases are among the enzymes able to cleave l-Leu-AMC. Cleavage after an alanine residue has also been observed for the serine peptidase elastase. Elastase can accommodate small and neutral side chains such as glycine, alanine, serine, and valine during the processing of cathelicidins, a type of AMPs [44].

There is a possibility that the tested secretion is able to cleave after the polar residue tyrosine (Y) and the basic residue arginine (R). Nevertheless, the peptides presenting these residues have their N-termini blocked, which might be disabling the proteolytic activity. To illustrate, the fluorogenic peptides N-Succinyl-Gly-Pro-Leu-Gly-Pro-AMC and N-Succinyl-Ile-Ala-AMC were not cleaved, while the substrates Gly-Pro-AMC and l-Alanyl-l-Alanyl-l-Phe-Ala-AMC liberated AMC when they were incubated with the sample. Despite the determination of preferential cleavage sites, some researchers have shown that the secondary structure of the peptides or neighboring amino acids plays an important role in determining the accessibility of the site to proteolysis [16], [18], [45].

### Influence of Protein Concentration on Substrate Hydrolysis

**Figure 4:**
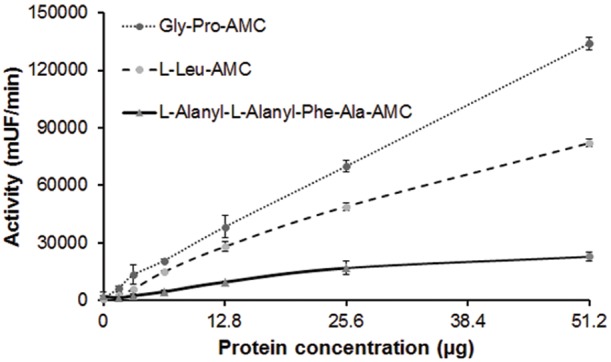
Influence of protein concentration of the crude skin secretions of *L. labyrinthicus* on the proteolysis (mUF/min) of three fluorogenic substrates: Gly-Pro-AMC; l-Leu-AMC, and l-Alanyl-l-Alanyl-l-Phe-Ala-AMC. The assay was performed in 25± standard deviation (SD). mUF/min = milli units of fluorescence liberated per minute.

The protein concentration of the sample was directly proportional to the proteolytic activity. This relationship was more prominent in the hydrolysis of Gly-Pro-AMC (Fig. 4). These results agree with those by Merchant et al. [37], who described that the enzymatic activity of American crocodile serum depends on the serum titer.

### pH Dependence of Fluorogenic Substrate Hydrolysis

**Figure 5:**
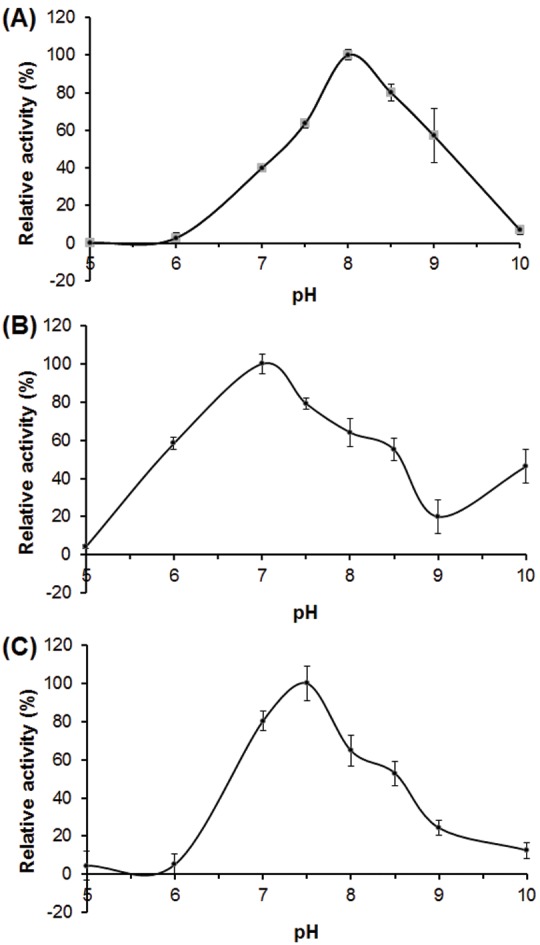
The pH dependence of the proteolytic activity present in the crude skin secretions of *L. labyrinthicus* on fluorogenic substrates. (A) Gly-Pro-AMC; (B) l-Leu-AMC; (C) l-Alanyl-l-Alanyl-l-Phe-Ala-AMC. The assays were performed in AMT buffer (100 mM acetic acid, 100 mM MES, and 100 mM Tris-base). The results are expressed as the percentage of the maximum activity obtained. Each point represents mean ± SD.

The pH profile of the proteolytic activity of the enzymes present in the sample was assayed using the three substrates described above, and the maximal enzymatic activity was obtained near physiological pH. The highest activity was observed at pH 8.0 for the hydrolysis of Gly-Pro-AMC (Fig. 5A), at pH 7.0 for l-Leu-AMC (Fig. 5B), and at pH 7.5 for l-Alanyl-l-Alanyl-l-Phe-Ala-AMC (Fig. 5C). The l-Alanyl-l-Alanyl-l-Phe-Ala-AMC hydrolysis was very sensitive to acidic pH conditions, showing complete lack of activity at pH 6.0 (Fig. 5C). Inhibition of Gly-Pro-AMC hydrolysis at low pH indicated that the secretion did not contain DPP2, which has a maximum activity at pH 5.0–6.0 and is not active at basic pH values [46], [47]. Likewise, l-Leu-AMC hydrolysis was higher at alkaline pH, with more than 50% activity at pH 8.5. Moreover, we registered an increased activity at pH 10.0, after the lowest hydrolysis rate at pH 9.0, suggesting the contribution of a second enzyme in l-Leu-AMC cleavage. The highest activity was observed at a neutral or slightly basic pH, which is in accordance with the pH value of 6.9 of the skin secretion of *L. labyrinthicus*. This pH is the value in which peptidases are naturally active. Moreover, this is the pH range in which proline-specific DPPs are active [47]. Tang et al. [48] showed that a human DDP9 had its maximal activity between pH 7.5 and 8.0. An optimum pH of 8.0 was also determined for a DDP IV isolated from the entornopathogenic fungus *Metarhizium anisopliae* [49]. In summary, pH profile analysis suggests the presence of different peptidases participating in the hydrolysis of the synthetic substrates, and even more than one enzyme cleaving the l-Leu-AMC peptide.

### Inhibition Profile of the Peptidases by Fluorogenic Assays

Fluorogenic assays were performed to determine the peptidase classes present in the skin secretion of *L. labyrinthicus.* The data showed that the metallopeptidase inhibitor phenanthroline completely inhibited Gly-Pro-AMC and l-Leu-AMC cleavage (Table 2). Although classified as serine protease, dipeptidyl peptidase 4 is not much sensible to PMSF and its inhibition by phenanthrolines was already noticed to a DPP4 like from bovine serum [50]. The hydrolysis inhibition of l-Leu-AMC by phenanthroline together with the optimal pH results (pH 7.0) suggested the presence of metallopeptidases. The release of leucine could have been the result of the activity of leucine aminopeptidases (LAPs). LAPs are metallopeptidases that cleave N-terminal amino acids residues, preferentially leucine, from protein and peptides [51], and have optimum activity at neutral/basic pH. A LAP with similar optimum pH profile, maximum activity at pH 7.0 and inactive at pH 5.0 and 9.0, has been purified from *Trypanosoma cruzi* [52]. However, EDTA and bestatin, other metallopeptidase inhibitors, did not inhibit the hydrolysis of this substrate. Similar results were observed by Berthonneau and collaborators [53], who described an immunogenic metallopeptidase that was inhibited by EDTA and *o*–phenanthroline but not by phosphoramidon. Gilmartin and O’Cuin (1999) purified an aminopeptidase from the cytoplasm of guinea-pig brain that was also completely inhibited by phenanthroline and only partially inhibited by EDTA [54]. These contradictory results may be explained by the fact that the studied secretion is a mixture of enzymes and not a pure peptidase. Phenanthroline was responsible for a 48% reduction of l-Alanyl-l-Alanyl-l-Phe-Ala-AMC hydrolysis, although the greatest inhibition of this peptide hydrolysis was observed with the serine peptidase inhibitor PMSF, which caused an approximately 80% inhibition. The serine peptidase inhibitor TLCK was responsible for 44% inhibition of l-Leu-AMC hydrolysis and 34% inhibition of l-Alanyl-l-Alanyl-l-Phe-Ala-AMC hydrolysis. PMSF also caused a 33% decrease of the proteolytic activity on Gly-Pro-AMC, despite not being inactivated by the other serine peptidase inhibitors (TLCK and TPCK). The cysteine peptidase inhibitor E-64 and the aspartic peptidase inhibitor pepstatin A did not cause a significant reduction of the proteolytic activity on the tested substrates. The complex inhibitory profile may be related to the ability of some inhibitors to link, in a nonspecific way, with the active site of some enzymes that is different from their target [55]. In conclusion, the inhibition results support the activity versus pH profile data because metallo and serine peptidases are more active at neutral to slightly basic pH values than other peptidases [56], [57].

**Table 2:** Effect of different inhibitors on the peptidases present in the crude skin secretions of *L. labyrinthicus* (25.6 µg) on the hydrolysis of Gly-Pro-AMC, l-Leu-AMC and l-Alanyl-l-Alanyl-l-Phe-Ala-AMC.

Inhibitor	Concentration (mM)	Relative activity (%)		
		Gly-Pro-AMC	l-Leu-AMC	l-Alanyl-l-Alanyl-l-Phe-Ala-AMC
Phenanthroline	10	0.0	0.0	51.8
EDTA	10	80.8	100.0	100.0
Bestatin	0.01	73.0	89.5	88.8
TPCK	0.1	81.5	94.6	81.4
TLCK	0.1	89.4	55.8	65.4
PMSF	1	66.8	85.4	11.4
Leupeptin	0.1	87.1	98.3	98.1
E-64	0.01	100.0	100.0	100.0
Pepstatin A	0.001	74.2	100.0	80.4
Control	–	100.0	100.0	100.0

The incubation was performed in 50 mM HEPES buffer, pH 7.5 for 45 min.

### In-gel Activity of the Skin-secreted Peptidases of *L. labyrinthicus*

**Figure 6:**
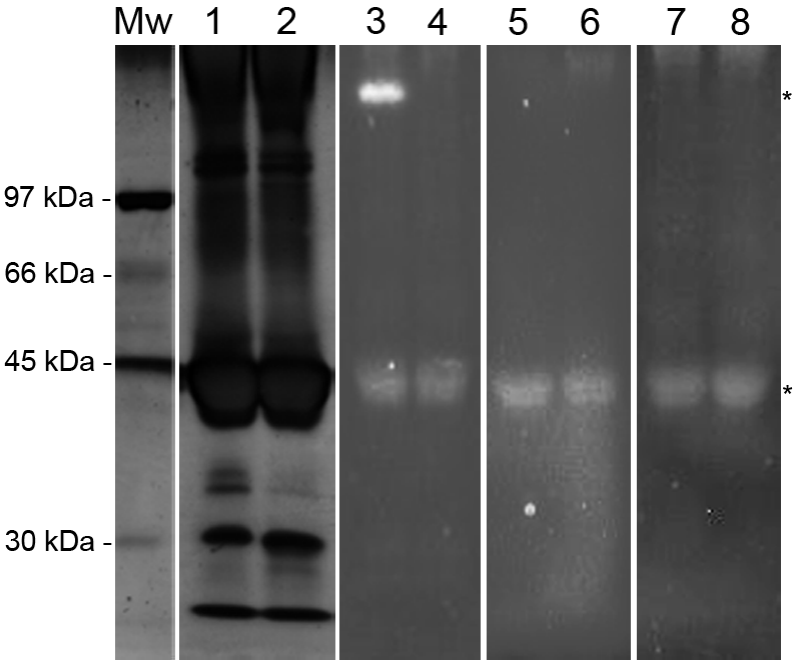
Proteolytic activity of the crude skin secretions of *L. labyrinthicus* (40 µg) on 9% SDS-PAGE incubated for 15 min with fluorogenic substrates. (Lanes 1 and 2) silver stained; (Lanes 3 and 4) Gly-Pro-AMC; (Lanes 5 and 6) l-Leu-AMC; (Lanes 7 and 8) l-Alanyl-l-Alanyl-l-Phe-Ala-AMC. Even numbers are boiled skin secretions incubated with the substrates. Mw: molecular weight markers.

The results of the 9% SDS-PAGE gels incubated with fluorogenic peptides showed that the 45 kDa band was responsible for peptide hydrolysis. The high abundance of protein at this molecular weight was revealed by silver staining (Fig. 6). After incubation with the substrate Gly-Pro-AMC, proteolytic activity was observed in a band near 200 kDa. However, when the sample was boiled, this proteolytic band disappeared, suggesting that this band may be (1) an oligomer of the 45 kDa protein, since the sample was not boiled and the sample buffer lacked a reducing agent; (2) a thermosensitive protease that lost its activity after the sample was boiled; (3) another enzyme, such as the dimer of DPP4 like enzymes, which lose their activities in their monomeric state (∼100 kDa) [58]. The presence of oligomers in the sample can be clearly visualized in the silver-stained gel (Fig. 6); after boiling the sample, the two bands of approximately 35 kDa almost disappeared. Nevertheless, these observations do not mean that the same enzyme was responsible for the hydrolysis of all substrates. It is possible that other peptidases of similar molecular weight are responsible for proteolysis, since the inhibition partner, pH profile, and reaction velocity were distinct to each substrate.

## Concluding Remarks

The peptidases present in the crude skin secretion of the pepper frog *Leptodactylus labyrinthicus* have different specificities or are broad-specificity peptidases. Until now, it was not found in the skin secretion of *Leptodactylus* truncated peptides derived from hydrolysis between the positions 1 and 11. Nevertheless, the number of peptides resulting from proteolytic processing is low; therefore, definitive conclusions regarding the occurrence and frequency of cleavage in the N-terminal region of peptides and the specificity of these cleavages cannot be made.

Hydrolysis of the peptide Gly-Pro-AMC suggests the presence of DPPs and/or metallopeptidases in the studied secretion. Nonetheless, DDPs would not be able to process large peptides such as AMPs. Thus, other peptidases may be involved in this process. If the presence of DPPs in the skin secretion of *L. labyrinthicus* is confirmed by future studies, it may reveal an interesting role of these enzymes. It is known that DPPs can cleave the neuropeptide bradykinin; and there is evidence that some DPPs are involved in immunoregulation and wound healing, which are the same functions played by the AMPs found in anuran skin secretions [47], [59], [60]. In addition, some AMPs are similar to neuropeptides and are bradykinin-like [5], [6].

Analysis of the enzymatic activity of anuran skin secretions is not very common, since the search for bioactive peptides or protease inhibitors is more frequent [61]–[63]. Giovaninni et al. [16] have suggested that the peptidases found in anuran skin secretions could be packed within the vesicles but inactive before secretion. The peptidase activity observed in the skin secretion of *L. labyrinthicus* may be involved in the regulation of tegument homeostasis and peptide processing, inactivating peptides harmless to the frog itself or generating peptides with novel functions. Since fallaxin fragments possess no antimicrobial activity, its processing in *L. labyrinthicus* suggests a mechanism to protect the frog against the cytotoxicity of the peptide. It was observed that the AMP citropin 1.1 is processed by endopeptidases 10 min after being released, eliminating its biological activity [64]. Furthermore, Resnick et al. [18] have shown that some peptide fragments undergo C-terminal amidation after proteolytic processing. In this way, the antimicrobial activity lost with hydrolysis can be restored [65], or the peptides may acquire a new function. On the other hand, there are reports regarding peptide fragments such as SPX(1–16) and SPX(1–22), derived from the AMP syphaxin, which display potent biological activity without any processing after hydrolysis [24].

To conclude, further research is necessary to better characterize the peptidases of this secretion and to elucidate their biochemical functions. Future studies may also identify new peptidases. The present work contributes to improve the knowledge regarding the peptidases present in anuran skin secretions and is the first time that enzymatic activity was studied in the skin secretion of the genus *Leptodactylus*.
